# Technology on Snow and Ice: Innovation, Monitoring, and Performance for the Olympic Winter Games Milano Cortina 2026

**DOI:** 10.1111/sms.70218

**Published:** 2026-02-07

**Authors:** Andreas Almqvist, Matej Supej, Peter Düking, Thomas Stöggl, H.‐C. Holmberg

**Affiliations:** ^1^ Division of Machine Elements Luleå University of Technology Luleå Sweden; ^2^ Faculty of Sport University of Ljubljana Ljubljana Slovenia; ^3^ Department of Sports Science and Movement Pedagogy Technische Universität Braunschweig Braunschweig Germany; ^4^ Department of Sport and Exercise Science University of Salzburg Salzburg Austria; ^5^ Red Bull Athlete Performance Center Salzburg Austria

**Keywords:** aerodynamics, data analytics, governance, performance, technology, tribology, wearables, winter sports

## Abstract

Elite performance in Olympic winter sports depends on the interplay among the athlete, equipment, and the snow or ice. This naturally evolves with temperature, humidity, wind, preparation, and contact between the equipment and its surface. Together, these factors continuously rebalance the forces of gravity, aerodynamic drag, and friction, requiring athletes, coaches, and organizers to adapt technique, equipment, and surface management, e.g., snow grooming and salting, ice resurfacing or pebbling, and rink climate control. This narrative review (SANRA‐guided) synthesizes the scientific literature across four domains: (i) the evolution of equipment and athlete–surface interaction; (ii) the physics of resistive forces and targeted countermeasures; (iii) sensing and monitoring with robust, field‐validated technologies and analytics; and (iv) the digitalization of coaching, officiating, and broadcasting. We integrate design and validation with sport regulations and governance. This includes the ban on fluorinated waxes, geometry and mass limits, and principles for data stewardship, model transparency, and fairness. A central component of this review is the assessment of quality aspects of technologies, including the assessment of ecological validity under field‐specific conditions before their use in high‐stakes coaching, medical, or officiating decisions. We conclude with actionable recommendations for Milano–Cortina 2026: (i) align equipment and surface preparation with expected regimes of drag and friction; (ii) deploy sensors and analytics with demonstrated accuracy, precision, and reliability; (iii) quantify uncertainty in key performance indicators; and (iv) treat federation rules as a priori design constraints. This approach enables innovation to deliver faster, safer, and more equitable outcomes in winter sport at Milano–Cortina 2026 and beyond.

## 1 Introduction

Technological innovation has become indispensable to high performance in Olympic sports, particularly for performance monitoring and managing environmental volatility [1, 2, 3, 4]. While summer disciplines often occur in controlled environments with stable surfaces, winter events unfold on deformable snow and ice surfaces. These surfaces evolve dynamically with temperature, humidity, and wind, as well as with equipment–surface interactions, depending on the state of preparation [5, 6]. This environmental volatility is a defining characteristic that distinguishes winter from summer sports, continuously rebalancing gravitational load, aerodynamic drag, and surface friction. Consequently, athletes must continuously adapt their technique, pacing strategies, and equipment selection [3, 4, 7, 8, 9].

Beyond these environmentally induced changes, the equipment–surface contact compacts and abrades snow, creating frictional melt films that alter the local microstructure and lubrication at the interface, dynamically shifting the frictional conditions athletes are facing [10, 11, 12]. Collectively, these environment‐ and contact‐driven changes define a distinct technological challenge where the primary objective is to manage the dynamic, condition‐dependent interaction between the athlete, equipment, and a constantly changing field of play. To address this complexity, new wearable sensor technologies claiming to provide actionable insights into training and competition are increasingly available, aiming to improve decision‐making and performance [13, 14].

**FIGURE 1:**
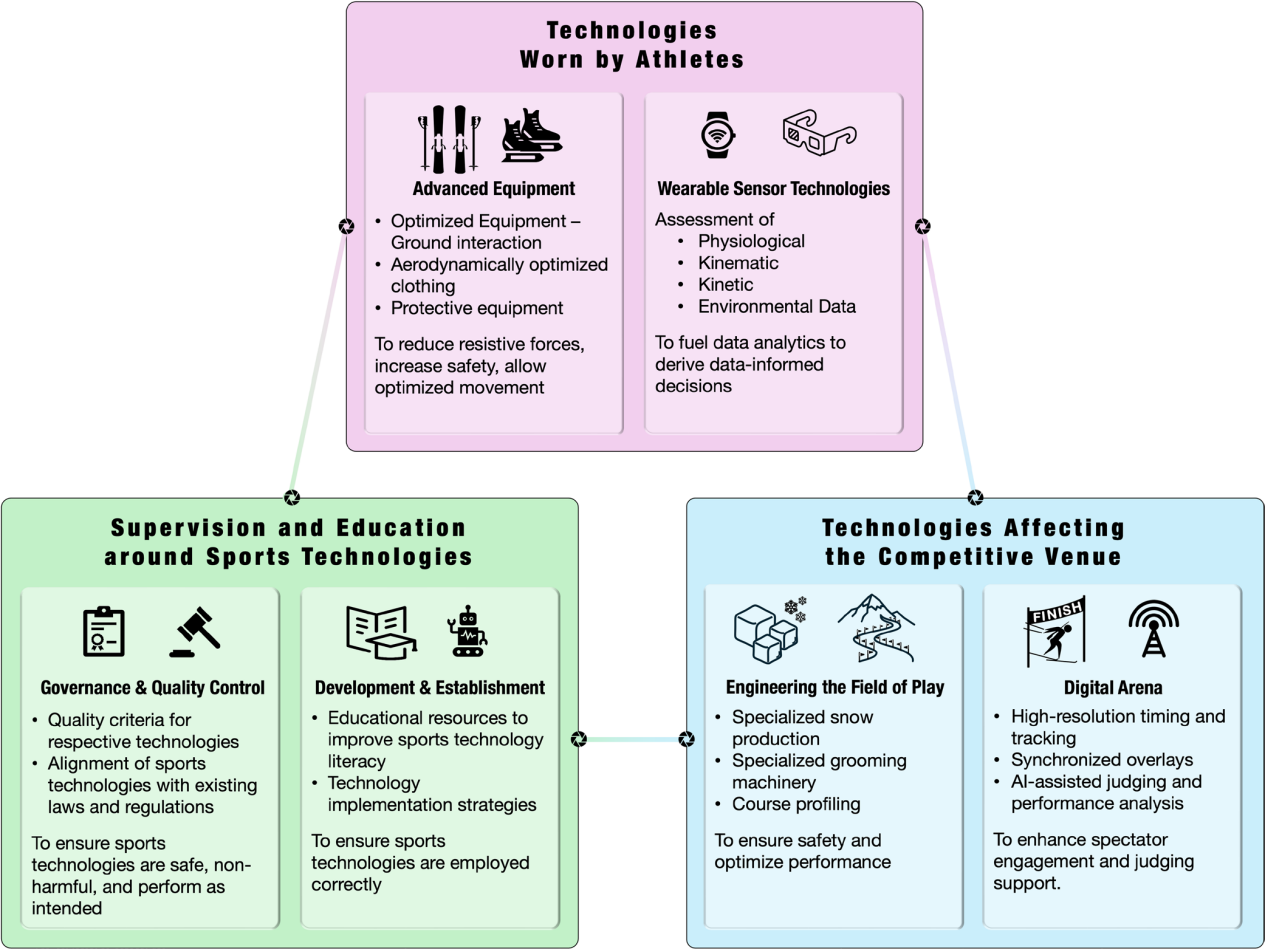
Overview of the technological ecosystem in Olympic winter sports, with three key domains of technological innovation interacting to reduce frictional forces, enhance safety, and enable data‐driven performance and fair competition.

Over the last two decades, a technological revolution (next to others, such as improvements in nutrition [15, 16]) has and will continuously shape the dynamic landscape of elite (winter) sports [17] as adoption rates of certain technologies are very high [18]. Advances in material science have yielded carbon‐composite ski constructions, instrumented curling brooms, and novel steel alloys for skate blades [4, 19, 20]. Concurrently, ubiquitous sensing technologies like Global Navigation Satellite Systems (GNSS) and Inertial Measurement Units (IMUs) have moved from laboratory research to integrated field applications [21, 22, 23]. The resulting high‐frequency, multimodal datasets increasingly feed analytic workflows, often incorporating edge computing and artificial intelligence (AI) to provide near real‐time feedback [24]. These advancements have fundamentally altered preparation and competition across endurance disciplines, high‐speed events, and arena‐based sports. An overview of this technological ecosystem and its major interacting domains is shown in Figure 1.

In parallel with athlete‐facing technology, a growing digital arena has transformed performance measurement, officiating, and spectator engagement [4, 22, 25, 26, 27, 28, 29, 30, 31]. Innovations such as broadcast overlays, automated judging support, and comprehensive object tracking have created a richer, more transparent experience for stakeholders from officials to fans [29, 32, 33, 34].

Critically, this wave of technological innovation does not occur in a vacuum. It is often bounded by strict regulatory frameworks set by international federations, like the International Ski and Snowboard Federation (FIS), International Skating Union (ISU), and International Bobsleigh & Skeleton Federation (ISBF). These regulations, governing parameters such as course preparation, non‐fluorinated wax composition, ski geometry, pole length, suit permeability, and sled mass, are designed to balance safety, fairness, and sustainability while permitting sport‐specific optimization [35, 36, 37, 38, 39, 40, 41]. Consequently, modern performance strategies must navigate the intersection of engineering potential and regulatory constraints, addressing emerging challenges in data governance, validation of predictive models, and equitable access to technology, thereby mitigating a wealth‐based performance gap [42].

Timed to support preparation for the Milano–Cortina 2026 Olympic Winter Games, this narrative review synthesizes state‐of‐the‐art scientific literature across the technological ecosystem of winter sports. We address four key domains: (i) the co‐evolution of equipment and athlete–surface interactions; (ii) the physics of environmental resistive forces and the technological solutions designed to mitigate them; (iii) the application of technologies to assess biomechanics and internal‐external load; and (iv) the rise of the digital arena, including AI in officiating and broadcasting, alongside associated governance and quality control requirements. Throughout this review, we map technology maturity across disciplines and propose directions for future research. The ultimate goal is to provide a practical resource that helps teams select, evaluate, and deploy technology that translates into effective, fair, and sustainable performance on snow and ice.

## 2 Methods

This manuscript is a structured narrative review, adhering to the Scale for the Assessment of Narrative Review Articles (SANRA) criteria to ensure transparency and quality. An a priori protocol was developed to prespecify the review's scope, research questions, and synthesis methods (not registered but recommended for inclusion as a supplementary file for transparency).

### 2.1 Search Strategy

A systematic search of the PubMed/MEDLINE, Web of Science, and Scopus databases was conducted for all literature from inception to July 31, 2025. The search strategy employed concept blocks combining keywords and MeSH terms for (1) Olympic winter sports (e.g., “alpine skiing,” “bobsleigh,” “biathlon”); (2) technology and environment (e.g., “equipment,” “innovation,” “wearable,” “sensor,” “snow,” “ice,” “friction,” “aerodynamics”); and (3) outcomes (e.g., “performance,” “biomechanics,” “physiology,” “injury,” “safety”). To ensure comprehensive coverage, backward and forward citation tracking was performed on all included articles. Recognizing that much of the cutting‐edge innovation in this applied field is documented outside of traditional academic journals, we also screened gray literature. This included federation rulebooks and technical documents, reports from national Olympic and sport science institutes, patents, and peer‐reviewed conference proceedings (e.g., from the International Congress on Science and Skiing).

### 2.2 Eligibility

Sources were included if they were peer‐reviewed studies, engineering analyses, consensus statements, or technical reports that addressed elite or near‐elite athletic populations. We also included mechanistic studies providing fundamental insights (e.g., tribology, material science) that were directly translatable to Olympic‐level performance. Studies focusing exclusively on youth or recreational cohorts, or those in purely clinical or rehabilitation contexts, were excluded unless they provided a unique insight directly applicable to high performance.

### 2.3 Appraisal and Synthesis

Almqvist and Holmberg independently screened titles, abstracts, and full texts against the eligibility criteria. The quality of included studies was appraised using design‐appropriate tools (e.g., a modified Downs & Black checklist for non‐randomized studies; AMSTAR‐2 for systematic reviews), with the assessed risk of bias used to weight the interpretation of findings. Due to the heterogeneity of the included sources, a meta‐analysis was not feasible. Instead, findings were synthesized thematically into the four core domains outlined in the introduction: equipment and materials, resistive forces, sensing and monitoring, and the digital arena with its associated governance.

## 3 Evolution of Equipment in Olympic Winter Sports

Equipment in Olympic winter sports has transitioned from simple constructs of wood, leather, and steel to sophisticated systems integrating advanced composite materials, complex geometries, and athlete‐specific tuning [2, 4, 20]. This evolution reflects an increasingly precise understanding of the biomechanics and physics governing performance on snow and ice [4, 5, 6, 20, 43].

### 3.1 Snow‐Based Sports

#### 3.1.1 Alpine Skiing

The evolution of alpine ski equipment has been characterized by innovations that directly altered the mechanics of turning [4]. Advances in sidecut geometry, evolving from conventional, weakly waisted skis to parabolic profiles with increased sidecut depth and decreased radius, combined with tailored longitudinal and torsional stiffness profiles, have enabled cleaner carving, greater edge grip, and higher speeds [44, 45, 46].

Modern skis utilize complex composite layups (carbon, fiberglass, titanal) around a wood core, with specific layers tuned to control stiffness and damping [46, 47]. Binding and plate systems have evolved into critical performance components, acting as mechanical interfaces that influence load transfer and ski bending under the boot while maintaining reliable retention and release [48]. Increasing stand height delays boot–snow contact and enables larger edge angles, but higher stand height also increases skier leverage and kinetic energy, prompting FIS to impose strict limits for safety [49, 50].

Designs diverge by discipline: slalom skis are shorter with tighter sidecut and high torsional stiffness for rapid edge reversals, whereas downhill skis are markedly longer with larger turn radii, greater mass, and added damping (e.g., plate/viscoelastic layers) to maximize stability at high speed [50, 51]. Boot technology has similarly progressed to stiff plastic shells with individualized alignment (cuff/canting, ramp angle), where precision fitting is now a critical determinant of snow feel [52, 53]. (For discipline‐specific personal protection and course safety systems, see “Protecting the Athlete”).

#### 3.1.2 Freestyle Skiing & Snowboarding

While sharing foundational materials and tuning principles with alpine racing, freestyle skiing, and snowboarding equipment are optimized for entirely different objective functions. In park‐and‐pipe, athletes favor softer longitudinal flex and tailored rocker/camber to enhance “pop” for take‐offs, improve maneuverability in the air, and provide more forgiving landings during complex aerial maneuvers [54, 55]. Twin‐tip geometries, symmetrical sidecut, and reinforced edges and bases improve switch skiing and durability on rails and boxes. Detuned contact points reduce edge‐catch risk on features, while binding stance width/angles and boot‐flex profiles are tuned for trick repertoire and landing stability [56].

Mogul skis, by contrast, prioritize torsional control and vibration damping to keep edge precision in the rut line. Their low mass enhances rapid absorption and directional changes, while relatively small sidecut radii enable fast edge engagement [57]. In snowboard cross (SBX) and parallel giant slalom (PGS), equipment shifts toward carving stability and effective power transfer. Boards with higher torsional stiffness, longer effective edges, and plate systems—combined with hard boots and plate bindings in PGS—support edge hold at speed and maximal acceleration off the start gate. By contrast, SBX setups adjust the stiffness–damping balance to cope with chop, absorb large jumps and rollers, and maintain reliable edge hold through the course [4, 49, 58].

#### 3.1.3 Ski Mountaineering

Debuting as an Olympic discipline at the Milano–Cortina 2026 Winter Games, ski mountaineering presents a unique engineering challenge: equipment must reconcile the competing demands of extreme lightness for ascents and robust control for technical descents [59, 60], while complying with International Ski Mountaineering Federation (ISMF) equipment rules specifying minima for ski length/width and a minimum ski‐plus‐binding mass [61]. Race execution relies on removable adhesive skins for uphill grip, minimalist pin bindings that permit free‐heel “walk mode” and rapid transitions, and cuff‐articulating, rubber‐soled boots that preserve uphill economy without compromising downhill safety [7, 62].

#### 3.1.4 Cross‐Country Skiing & Biathlon

Contemporary skis feature carbon‐ and glass‐fiber sandwich constructions around lightweight cores (e.g., honeycomb Nomex) on sintered ultra‐high‐molecular‐weight polyethylene (UHMWPE) bases [63]. The performance depends on physical properties of the ski, such as stiffness, camber shape, and the material and topographic features of the base, which significantly influence the performance [64, 65]. The base is precisely tuned via stone grinding and rilling to manage the thin water film between the ski base and the snow surface, governing glide friction, minimizing losses from compaction, abrasion, and direct ski–snow contact [5]. Skating ski side cut design has also varied, with manufacturers experimenting with straight, arrow‐like (getting smaller from tail to tip), tailed skis (e.g., Atomic Gen S; [66]) or negative tailed (Fischer skate cut) with a broader mid‐part of the ski.

Elite skiers have fleets containing multiple pairs of skis whose camber and bending stiffness are measured and matched to their body mass and force application, in order to balance classic grip–glide and skating push‐off. Pole technology has likewise advanced: high‐modulus carbon shafts have replaced aluminum to improve stiffness‐to‐weight and pendulum properties for energy transfer [3, 67, 68, 69]. With only a few exceptions, manufacturers design their top‐tier pole shafts with identical geometry and carbon–composite layup across lengths [70]. Consequently, both the pendulum properties and effective stiffness are dependent on shaft length, rather than being length‐independent design parameters.

To preserve classic technique and discourage exclusive double‐poling without grip wax, the International Ski and Snowboard Federation (FIS) limits classic pole length to 83% of body height; this height‐scaled cap alters available leverage, making athlete anthropometry and upper‐body strength critical factors in pole selection [36]. The MODD “Air Grip” is one commercially available example that uses a hinged lever to increase the effective pole length during the final part of the push‐off. This temporary pole‐length extension is intended to improve propulsive mechanics, yet no independent scientific validation of any performance effects is available [71].

In biathlon, equipment optimization extends beyond skis and poles to rifle ergonomics, where stock fit, sling geometry, and trigger characteristics are tuned to enable a stable sight picture immediately after high‐intensity work [2]. Under International Biathlon Union (IBU) rules that fix the caliber (0.22 LR) and define key rifle constraints, athletes optimize stock fit, sling geometry, and trigger setup for a stable sight picture immediately post‐effort [2, 72]. This enables rapid yet precise shot sequences in cold conditions while maintaining control with gloves.

#### 3.1.5 Ski Jumping

In ski jumping, equipment functions as an integrated aerodynamic system, precisely engineered to maximize flight distance within a framework of stringent regulations [73, 74, 75]. Ski length is scaled to body height but capped at 145% of body height, conditional on a minimum Body Mass Index (BMI) [36]. Jumping suits must meet the current FIS specifications for panel mapping, seam placement, size tolerances, permitted stretch, and minimum air‐permeability, with compliance verified through pre‐start and in‐competition controls [36, 76]. Within these constraints, equipment characteristics—including ski stiffness and boot‐alignment settings—must support a stable in‐run posture and accurate body–ski geometry, which are essential for effective take‐off timing [74, 76, 77]. Bindings serve as the critical interface for power transmission, ski‐to‐boot positioning during air time, and safety release [78]. They must be mounted so that no more than 57% of the total ski length is in front of the boot toe. Additionally, the combined height of the boot sole and any external wedges is regulated [36].

### 3.2 Ice‐Based Sports

#### 3.2.1 Long‐ and Short‐Track Speed Skating

In ice‐based sports, equipment and technique have co‐evolved, each constraining and enabling advances in the other. Nowhere is this clearer than in speed skating [79]. The most iconic innovation, the *clap skate*, decouples the blade from the heel. This allows the blade to maintain ice contact during the terminal push‐off, permitting natural ankle plantarflexion and improving work transfer, which drove marked drops in world records [20, 80].

Modern performance relies on an integrated boot–blade system featuring thermoformable carbon‐composite boots. These are typically custom‐molded using a negative cast or 3D foot scan to ensure the stiffness required for force transmission during deep crouches [81]. Blade rocker and bending stiffness are matched to athlete mass and technique to maintain ice contact and cornering stability [80, 81]. Edge geometry, specifically the sharpening angle, radius of hollow, and longitudinal curvature, governs penetration depth and cut‐in, trading off maneuverability and glide speed [82]. Suit design has likewise become an engineering lever, with seam placement and surface texturing aimed at delaying separation and reducing drag [20, 83].

Long‐track and short‐track speed skating impose different equipment solutions. In long‐track, skaters use clap skates (hinged heels) and ultra‐stiff boots to sustain force application at high speed on comparatively open curves. In short‐track, clap mechanisms are prohibited, and athletes rely on longer, pre‐bent blades that are mounted rigidly, usually offset toward the inside (left) to improve grip in tight turns and high lean angles. Cut‐resistant suits, neck guards, and reinforced gloves are required because of pack dynamics and collision risks [84, 85].

#### 3.2.2 Bobsleigh, Skeleton & Luge

In sliding sports (bobsleigh, skeleton, luge), performance is determined by millimeter‐scale tolerances and millisecond‐level timing. Optimization focuses on three key areas: distributing mass up to regulatory maxima (often via ballast), minimizing aerodynamic losses through cowling design and athlete posture, and tuning runner steels (via selecting, profiling, and polishing) to match track‐specific ice conditions [27, 86, 87]. For luge, runner performance depends on steel–ice shear and real contact area, which vary with temperature and are modified by steering‐induced and wind‐induced vibrations [88]. Start performance is engineered via spike patterns and handle design to maximize impulse while preserving body–sled alignment during the loading phase [86, 89]. Within tight rule sets, teams iterate runner radii and finish quality to tune shear vs. plowing resistance along the track [89].

#### 3.2.3 Ice Hockey

Carbon‐composite sticks with tuned kick points (low/mid/high) and torsional profiles allow rapid release while maintaining puck control. Flex and length are selected relative to player strength, stature, and shooting style, with shaft and torsional stiffness affecting wrist‐shot velocity and release dynamics [90, 91, 92, 93]. A systematic review cataloging on‐ice testing protocols provides a framework for selecting valid performance metrics [94]. Custom‐fit, heat‐moldable skate boots enhance power transfer and comfort, while quick‐change holders and profiled blades (rocker and radius of hollow) allow on‐venue adaptation to ice hardness and tactical demands. The influence of blade geometry on friction and metabolic cost has been quantified [95, 96]. Equipment choices remain bounded by International Ice Hockey Federation (IIHF) equipment rules for sticks and skates [97]. (Protective systems and rink‐side safety engineering are detailed in “Protecting the Athlete”).

#### 3.2.4 Figure Skating

For figure skating, the boot–blade interface is critical for executing complex jumps and spins [98, 99]. Boot stiffness must be matched to athlete mass and jump repertoire to provide adequate ankle support without impeding the range of motion essential for explosive take‐offs; heat‐moldable shells and custom fitting (via focused heat shaping and brand‐specific lasts) are now standard at the elite level [100, 101]. Blade geometry, radius of radius, rocker profile, and toe‐pick configuration are individually tailored to balance edge bite against effective contact length, stabilizing jump entries and landings while supporting spin centering and axis control [83, 96, 102]. Wear‐resistant steel and finishing help preserve sharp edges through repeated jump impacts, while small changes in mounting alignment measurably alter lower‐limb kinematics, supporting the practice of minimizing torsional lag at the boot–blade interface for multi‐rotation jumps [103, 104]. Recent instrumented‐blade work further demonstrates that on‐ice loading patterns can be quantified directly, underscoring the sensitivity of blade mechanics to mounting and alignment [105].

#### 3.2.5 Curling

Even the seemingly traditional sport of curling is driven by advanced materials engineering. Precision‐machined granite stones (with tightly controlled running bands) interact with a purpose‐built pebbled ice surface [106]; brush‐head textiles and shaft stiffness are engineered to modulate friction and curl by altering pebble wear and local melt dynamics [107, 108, 109]. Following the 2015 “broomgate” episode, the World Curling Federation (WCF) standardized brush‐head materials and constructions to preserve fairness, with updates for the 2025–2026 season [110, 111, 112]. In practice, carbon‐fiber handles and compliant heads are tuned for pressure and stroke rate, while instrumented brooms quantify sweeping force/pressure, frequency, and sweep angle to guide technique [19, 109, 113]. Across elite venues, water purity, pebble height (“nipping”), and climate set‐points are maintained within narrow bands to stabilize stone behavior [108, 114, 115, 116]. Parallel work uses machine‐learning models to predict curling‐stone trajectories and inform data‐driven decision‐making [117].

Collectively, the current state‐of‐the‐art in ice sports reveals a convergent paradigm: integrated athlete–equipment systems, tuned within stringent rulebooks, to optimize steel–ice or stone–ice contact mechanics, posture‐dependent aerodynamics, and durability under event‐specific constraints.

## 4 Environmental Resistive Forces: Gravity, Aerodynamic Drag, and Friction

Performance in winter sports is fundamentally a problem of energy management, specifically the athlete's ability to produce propulsive forces that overcome three primary environmental resistive forces: *gravity*, *aerodynamic drag*, and *friction*. Understanding and mitigating these forces is a primary objective of technological innovation.

### 4.1 Gravity

Course profiles, with their varying slopes and curvatures, define the potential and kinetic energy envelopes that an athlete must manage [21, 118, 119, 120]. While gravity's magnitude g is constant, its slope‐parallel component

$$F_{g∥}=\mathrm{mg}\sin\theta ,$$

varies with body mass m and slope θ, thereby determining acceleration on descents and resistive load on climbs. In downhill and sliding disciplines, this component is the principal propulsive term, and performance is a matter of minimizing energy dissipation [119]. Moreover, athletes optimize the trajectory taken from start to finish (“the line of skiing”), trading a shorter path against speed preservation through wider radii and reduced steering losses [4, 118, 120].

In endurance events like cross‐country skiing and the biathlon, $F_{g∥}$ becomes the primary form of resistance during uphill segments. This dramatically amplifies the metabolic cost and forces strategic selections of technique (e.g., the skating gears 2 vs. 3; double poling vs. diagonal stride vs. herringbone technique) [121, 122, 123] and pacing [124]. Technological solutions here involve course design and the use of lightweight equipment to reduce the system's mass that must be moved against gravity.

### 4.2 Aerodynamic Drag

As velocity increases, aerodynamic drag quickly becomes the dominant resistive force in high‐speed winter sports. The drag force, $F_{D}$, scales with the square of velocity, and the power, $P_{D}$, required to overcome it scales with the cube. That is,

$$F_{D}=\frac{1}{2}\rho C_{D}Av^{2},\;P_{D}=\frac{1}{2}\rho C_{D}Av^{3},$$

where ρ is the air density, $C_{D}$ is the drag coefficient, A is the frontal area, and v is the speed of the skier relative to the speed of the air. Because of this, drag largely governs top‐end speed in alpine skiing (on fast, steep sections), speed skating, and sliding sports [87, 125, 126, 127]. Consequently, techniques and technologies in these disciplines target drag reduction (posture, suits, helmets, and surface interactions). Athletes refine crouch postures in wind tunnels, and equipment such as suits and helmets is engineered to reduce the drag force (e.g., smooth seams, texture control). An optimized alpine posture can reduce effective drag by more than 20%, translating to meaningful time gains over a downhill course [127, 128, 129].

Computational fluid dynamics (CFD), often coupled with wind‐tunnel tests and 3‐D body scans, is now routinely used to simulate athlete–equipment flow fields and iterate design before on‐snow/on‐ice testing [127, 130, 131, 132]. For female ski jumping athletes, BMI‐linked ski length and suit‐fit rules constrain aerodynamic exploitation; consequently, tuning ski stiffness and boot alignment can aid in‐run stability and precise take‐off in lower‐mass systems [36, 133].

### 4.3 Friction

The most complex and variable resistive force is friction, which occurs at the interface between the equipment and this snow or ice surface [6]. This is not the simple dry friction taught in introductory physics, but rather a complex tribological system in which multiple mechanisms act in parallel. Depending on snow or ice state and the load, friction may arise from adhesive shear in microscopic contacts, viscous drag within quasi‐liquid or meltwater films, compaction beneath the ski or skate, abrasion by the ski‐base texture or the skate blade, and capillary forces associated with liquid bridges [82, 134, 135, 136].

The contribution from *adhesion* may be described as

$$F_{\mathrm{adh}}=\tau A_{r},$$

where τ is the shear strength of the interface, and $A_{r}$ is the real area of contact, commonly approximated as $A_{r}\approx N/H_{\mathrm{ice}}$, where N is the normal force and $H_{\mathrm{ice}}$ is the hardness of ice [137, 138]. The contribution from *viscous drag* may be modeled by

$$F_{\text{visc}}=\eta A_{w}v/h,$$

where η is the dynamic viscosity of the melt‐water film, h its thickness, and $A_{w}$ the wetted area ($A_{w}\approx A_{r}$ [138]). The “quasi‐plastic” *compaction* associated with snow contact may be described as

$$F_{\text{comp}}=\mathrm{dN}/l,$$

where d is the compaction depth and l the apparent contact length [137]. A simplistic model describing the *abrasion* by the ski‐base texture or the skate blade reads

$$F_{\mathrm{abr}}=A_{\mathrm{pr}}H_{\mathrm{ice}},$$

where $A_{\mathrm{pr}}$ represents the real contact area projected in the sliding direction [136]. The friction force arising from capillary forces associated with liquid bridges is not as simply formulated [134].

The relative contribution of the aforementioned mechanisms shifts dynamically with temperature, humidity, velocity, and grooming, making friction on snow and ice inherently multi‐scale and condition‐dependent [139, 140]. The coefficients can also vary widely, from values < 0.005 on optimized speed‐skating ice [141] or in icy ski–snow conditions [142], to > 0.04 in cold, dry snow [65].

In skiing, the resulting resistive force is determined by the integrated effect of the skier's loading pattern and technique, the ski's capacity to distribute pressure dynamically across its glide zones, and the material properties, structure, and preparation of the ski base [65, 143]. This continuously interacts with snow state parameters such as density, crystal morphology, temperature, humidity, and grooming. Accordingly, the goal of ski preparation, through stone grinding, rilling, and the now fluor‐free waxing, is to balance all major frictional contributions, mitigating adhesion, viscous drag, compaction, abrasion, and capillary effects to achieve optimal glide [139]. A unique aspect of the classic technique is balancing grip and glide to achieve an efficient diagonal stride with minimal drag while performing the other sub‐techniques [64, 123, 144]. Grip is provided by a tuned grip pocket (hard wax/klister or mohair skins), selected based on snow temperature and crystal state; current rules require fluor‐free systems [63, 64].

In ice sports, friction is tuned primarily through blade geometry and ice preparation. The skate blade's radius of hollow, sharpening angle, rocker profile, and steel hardness together determine penetration depth, effective contact length, and the balance between edge bite and glide, while resurfacing techniques and ice temperature control ice hardness and the thickness of quasi‐liquid or meltwater films on the surface [82, 140, 141]. Across speed skating, short track, figure skating, and ice hockey, these parameters are adjusted to trade maximal glide against stability in cornering, jump landings, or rapid direction changes.

## 5 Engineering the Field of Play: The Technology of Snow and Ice Surface Preparation

While often taken for granted, the surfaces on which Winter Olympic events are contested are themselves highly engineered technological systems. The management of snow and ice is a performance technology that mitigates environmental volatility and stabilizes or intentionally shapes the resistive forces athletes experience, creating fair, safe, and durable fields of play.

### 5.1 Snow Venues: Production, Grooming, and Course Profiling

#### 5.1.1 Snow Production and Microstructure

Machine‐made snow typically exhibits denser, harder, and more rounded grains than fresh natural snow [145, 146]. This microstructure improves durability under repeated athlete passage but alters frictional and mechanical behavior, requiring surface‐preparation strategies tuned to expected liquid‐water content (LWC), crystal form, and temperature. The tribological link between grain morphology, water‐film thickness, and base micro‐topography explains why grind/rill/wax packages must be matched to the prepared surface [139].

#### 5.1.2 Grooming Machinery and Objectives

Modern groomers use tillers, finishers, renovators, and compactors to compact the snowpack, mill the surface to a target texture, and homogenize near‐surface density. On steep slopes, cable‐winch grooming is deployed to maintain consistent preparation [147].

Operationally, venues aim for a predictable coefficient of friction and sufficient near‐surface shear strength to limit rutting and friction drift. These aims are explicit in cross‐country grooming manuals and FIS course‐ and venue‐design guidance, which emphasize consistent surface quality, bearing capacity, and grooming schedules aligned to start times [148, 149]. Across disciplines, modern preparation has contributed to more consistent and fairer snow conditions, improved performance, and safety.

Discipline‐specific targets:

- *Alpine & Snowboard Parallel Giant Slalom:* Surfaces are prepared to “race‐grade” hardness to resist edge penetration and preserve the line through gates, standardizing track hardness across start groups. The method for achieving this race‐grade hardness depends on the temperature: under subfreezing conditions, local water injection or repeated mechanical compaction is used, whereas in warm (near‐melting) conditions, the application of salt achieves the same outcome [35, 147].
- *Freestyle Park & Pipe/Moguls/Snowboard Cross:* Preparation balances hardness for shape retention with a compliant top layer for edge purchase (reliable edge penetration and grip) and landings; features (kickers, transitions, rail decks) are machine‐shaped to centimeter tolerances and hand‐finished to ensure geometric consistency run‐to‐run [150, 151].
- *Cross‐country skiing & biathlon:* Courses are prepared to ensure optimal track quality across both techniques and all terrain segments. Grooming is timed and calibrated to prevailing snow and weather conditions and to start schedules, with the aim of preserving the highest possible track quality for athletes throughout the competition, reducing friction‐drift and rutting so waves and start orders encounter similar conditions [5, 149].

#### 5.1.3 Chemical Hardening (“Salting”)

As the near‐surface snowpack becomes isothermal at or near 0°C, intergranular bonding weakens and bearing capacity declines [35, 152, 153]. Controlled application of salts (e.g., NaCl/CaCl_2_) or nitrogen‐based agents (urea, ammonium nitrate/sulfate) can induce localized melt–refreeze that stiffens the top layer and stabilizes ruts; its effectiveness scales with snow temperature and liquid water content [35, 154]. Event manuals specify base preparation, that is, how to compact, smooth, and if glazed, lightly scarify/pre‐granulate the top layer; then conduct small‐area tests, apply salt evenly with mechanical spreaders, and allow a freeze‐in window before use. They also provide indicative dosing/timing relative to forecast and start order (e.g., ≈40 kg·km^−1^ in cross‐country skiing trials) [35, 152]. Owing to environmental concerns (chloride runoff, nutrient loading), national agencies regulate use; best practice is minimal, corridor‐limited dosing, mapping/avoiding sensitive zones, logging quantities, and post‐event remediation [153, 155].

#### 5.1.4 Course Profiling and Homologation

High‐resolution digital terrain models are generated using airborne/terrestrial LiDAR (vertical accuracy ≈2–5 cm, ≈5–10 cm horizontal). Real‐Time Kinematic (RTK) GNSS, and differential GNSS (dGNSS) (≈1–2 cm horizontal, ≈2–3 cm vertical) provide centimeter‐scale digital terrain models for homologation and course setting; differential GNSS verifies prepared geometry. Coupled with athlete trajectories, these models quantify how line choice and course geometry expose skiers/riders to aerodynamic and frictional costs at the element level, influencing their performance [21, 22, 29, 118]. In gate sports, offset, vertical, and rhythm are tuned to manage speed and ground‐reaction forces while preserving fairness and safety envelopes [156, 157].

### 5.2 Ice Venues: Making and Maintaining Fast, Durable Ice

#### 5.2.1 Long‐ and Short‐Track Speed Skating

Performance hinges on a thin, hard, and uniform ice sheet. Technicians rigorously control brine and air temperatures, humidity, and resurfacing‐water purity (often deionized/reverse‐osmosis) to limit impurities and micro‐roughness. Cooler, cleaner, smoother ice reduces viscous plowing and squeeze‐film losses at the steel–ice interface [82, 141].

At the Olympic Winter Games in Beijing 2022, transcritical CO_2_ direct‐cooling was implemented to improve heat‐transfer efficiency and spatial temperature control, contributing to uniform, fast ice [158, 159]. In parallel, rink‐operations guides emphasize controlled air/ice temperatures, humidity management, and high‐purity resurfacing water to reduce dissolved solids and micro‐roughness [160].

#### 5.2.2 Curling

Technicians engineer a purpose‐made surface on a laser‐leveled base using purified water, with pebbling (spraying fine droplets to form hemispherical asperities) and “nipping” (clipping the peaks) to set the topography that governs stone running and curl. Arena climate (air/ice temperature and relative humidity) is held within narrow bands, as are water purity and pebble height, to stabilize stone–ice interaction and sweeping effects across a session [30, 108, 116].

#### 5.2.3 Ice Hockey and Figure Skating

Hockey operations bias sheet temperature and resurfacing cadence toward higher hardness and low snow production to preserve glide and stable puck roll in heavy traffic; between‐period maintenance targets ruts along boards and creases, and restores planarity [161]. Figure‐skating sessions are often run slightly warmer and more compliant to enhance edge purchase and cushion jump landings, with element‐zone micro‐repairs (e.g., filling toe‐pick divots) performed alongside federation‐guided set‐points [83, 162].

#### 5.2.4 Sliding Tracks for Bobsleigh, Skeleton, and Luge

Tracks are refrigerated concrete channels on which ice is built and sculpted. Track crews manage longitudinal temperature gradients, corner ice thickness, and surface roughness to control speed, steering sensitivity, vibration, and safety margins [27, 163]. Nightly shaving/spritzing restores planarity, while localized glazing polishes race lines. Preparation balances low friction with predictable steering response to minimize energy loss and instability [164].

#### 5.2.5 Monitoring and Quality Assurance

Instrumented venues, with embedded thermistors in the ice/snowpack, hardness/penetrometer profiling, and surface roughness/conductivity probes, paired with weather stations, GNSS–video overlays, and post‐resurfacing audits, allow organizers to verify that preparation matches homologation targets and to detect drift that could alter friction or safety margins [29, 37, 163]. For snow surfaces, rapid penetrometers are established tools for near‐surface hardness/stratigraphy [165]. In arenas, federation guides specify continuous monitoring of ice/air temperature, humidity, and dew point to stabilize glide and reduce hazards [116, 160, 161].

#### 5.2.6 Sustainability and Regulation

Preparation choices operate within evolving regulatory and environmental constraints. Federation rulebooks and homologation documents define acceptable surface states and methods; recent fluor‐wax bans with in‐venue screening have shifted materials toward lower‐impact options [166]. Use of chemical snow hardeners is governed by formal guidelines in cross‐country, emphasizing conditions, dosages, and decision trees to ensure fairness and limit environmental load [35]. Venues increasingly optimize refrigeration energy usage in refrigeration and snow making, dehumidification, and water purification/reclaim loops, with sustainability guidance published by international federations [161, 164].

Collectively, these practices treat the venue as a controllable technological layer. Pre‐event plans should integrate snow/ice physics, forecast‐driven decision trees (groom vs. salt vs. resurface), measurement protocols, and clear communication to teams so equipment and pacing choices can align with the evolving “arena.”

## 6 Protecting the Athlete: Safety Technology in Olympic Winter Sports

Winter Olympic sport places athletes in high‐speed competition on hard, low‐compliance surfaces where high kinetic energy and minimal margins for error create significant injury potential.

Compared with many summer disciplines, typical operating speeds are higher, surfaces are harder, and (lower) temperatures make materials more brittle, all of which amplify impact severity. As a result, technology for injury prevention and mitigation is not peripheral but integral to performance [45, 167, 168].

### 6.1 Snow‐Based Sports

#### 6.1.1 Alpine Skiing, Freestyle Skiing & Snowboarding

Personal protection has evolved from simple hard shells to integrated systems that manage both linear and rotational loads [169]. Certified helmets with rotational‐energy mitigation, back protectors, shin/forearm guards, and hand protection (technical events), and, particularly in speed events, cut‐resistant suits and inflatable air‐bag vests that deploy within milliseconds during high‐energy crashes [168]. Governed by international standards [170, 171] for assembly, adjustment, release, and retention, boot–binding systems are engineered to release under complex, multi‐axis loads while ensuring effective load transfer [48, 172].

Beyond personal protection, FIS defines discipline‐ and sex‐specific minima for ski length/width and sidecut radius, plus a maximum standing height for the ski–plate–binding unit [49]. These constraints are intended to curb excessive leverage and carving capability, thereby improving stability, safety, and comparability [45]. Race suits are likewise standardized: Alpine suits must meet a minimum air‐permeability (30 L·m^−2^·s^−1^), uniform porosity, and carry the official conformity label (“CS 2015”), prohibiting surface treatments that would alter aerodynamic or permeability properties [49].

Specific technologies are used to alter the field of play in alpine, freestyle, and snowboarding competitions. For example, winch grooming, water injection, and salting deliver more uniform shear strength to limit rut growth. In addition, energy‐absorbing A‐/B‐netting, padding of fixed objects, and calibrated gate panels reduce impact severity. In freestyle and park disciplines, feature design (take‐off geometry, landing angle, snow density) and impact‐attenuating landing decks are engineered to lower deceleration loads while preserving performance [173, 174].

#### 6.1.2 Cross‐Country Skiing

Injury patterns in cross‐country skiing are predominantly caused by overuse. However, serious acute trauma can also occur from high‐speed descents, pole‐plant errors, and collisions (particularly in mass starts) that can cause serious trauma [175]. For this reason, a short neutral double‐poling start zone is prescribed to improve safety in mass‐start skate races [149]. Protective strategies are therefore dual‐purpose: reducing both impact injuries and chronic load, with a bias to the latter [176].

Pole straps and grips have evolved to optimize force transmission while minimizing wrist and thumb injuries [177]. Novel concepts, such as grips that transiently increase pole length, have been proposed, but peer‐reviewed data on their performance or injury implications remain limited [70, 71]. Ski‐pole baskets/ferrules are being developed to improve ground reaction and reduce slippage while treadmill skiing or on firm or icy tracks [69]. The shoe–ski interface (including sole stiffness and binding systems) must balance power transfer with ankle stabilization, and any novel grips or pole systems must comply with FIS equipment provisions (may not include artificial heating devices, chemical energy accumulators, electric batteries, mechanical aids, etc.) [36].

Given the cold environment, safety equipment and clothing must protect against frostbite while allowing freedom of movement, leading to the development of multilayered, windproof textiles especially to protect exposed areas such as the face and hands. Respiratory protection is also increasingly relevant, as many skiers report exercise‐induced respiratory symptoms in cold weather despite a low prevalence of clinical asthma or prolonged cough [178]. Emerging wearables, such as inertial sensors embedded in skis, may help monitor cumulative mechanical load and identify high‐risk patterns before injury develops [179]. In FIS Cross‐Country, the Jury applies specific precautions between −15°C and −25°C (e.g., wind‐chill assessment, course/format adjustments). At −25°C or colder, competitions must be delayed or canceled [180].

#### 6.1.3 Ski Jumping

Ski jumping presents a unique set of safety challenges, with athletes exposed to high speeds during in‐run, aerodynamic instabilities during flight, and significant impact forces at landing [41]. Although rare, serious injuries involve the knee, ankle, or spine, particularly during unstable or asymmetrical landings [133]. Surveillance across FIS World Cup seasons in elite women has further identified crash landings as the dominant mechanism and knees as the most frequently injured joint, including anterior cruciate ligament involvement [181]. This finding is consistent with biomechanical studies suggesting that subtle differences in ski position during the flight‐to‐landing‐transition can significantly affect load symmetry and injury risk [78].

Personal protective equipment is strictly regulated by FIS and includes helmets, binding release systems, and suits with controlled air permeability and stretch, all designed to ensure fair competition and safe aerodynamic behavior [36]. Equipment specifications are closely monitored, as violations can affect both performance and safety [74]. To reduce the risk of over‐rotation and unstable landings, jump hill profiles are engineered with carefully optimized take‐off angles, landing slopes, and transition zones [133]. Smooth grooming and consistent snow hardness across the landing area are essential to reduce local shear impact and enhance ski–snow adherence [133].

Emerging sensor technologies, such as IMUs, are increasingly being used in elite training environments to analyze in‐run stability, flight posture, and landing symmetry, supporting injury prevention and fine‐tuned feedback loops [77, 78]. They are not currently regulated for athlete use during competition under FIS Ski Jumping equipment rules; any in‐competition deployment would require formal approval and a standardized protocol. These systems may nevertheless play a future role in real‐time safety monitoring and landing risk detection.

### 6.2 Ice‐Based Sports

#### 6.2.1 Long‐ and Short‐Track Speed Skating

Pack racing at high speeds creates a distinct risk profile, combining lacerations from skate blades with high‐energy collisions into rink infrastructure. Consequently, International Skating Union (ISU) regulations [84] rigorously codify protection, mandating cut‐resistant apparel and helmets conforming to specific impact standards [182]. Rink‐side safety is addressed through regulations governing the technical performance and deployment of zoned padding systems [84]. For events with pack dynamics, such as the long‐track mass start, additional provisions are in place to mitigate collision hazards [183]. Despite this robust regulatory framework, peer‐reviewed evidence for the real‐world effectiveness of specific interventions remains limited, highlighting the need for standardized injury surveillance and systematic evaluation of protective apparel and barrier systems [184].

#### 6.2.2 Bobsleigh, Skeleton & Luge

Athlete protection in sliding sports requires an integrated safety system that couples personal equipment with sled and track engineering. High‐stiffness helmets with visors, neck collars, and abrasion‐resistant suits protect the athlete, while sleds are designed with controlled deformability and anti‐snag fairings to reduce secondary impacts [37, 38, 163]. Risk is further mitigated through carefully optimized track design, including corner radii, sightlines, compliant dasher profiles, and high‐yield boards backed by energy‐absorbing liners. Continuous monitoring of ice properties and local microclimate enables real‐time safety adjustments, such as modified start orders or heat delays under adverse conditions [27, 163].

#### 6.2.3 Ice Hockey

Due to the prevalence of rapid directional changes and collisions, protection in ice hockey is paramount. Modern helmets now combine multi‐density foams with rotational‐impact management certified to current standards (e.g., CSA Z262.1‐15), while comparative test frameworks such as Hockey‐STAR explicitly incorporate both linear and rotational kinematics [185, 186]. Shoulder and chest protectors have trended lighter without compromising coverage [97]. Cut‐resistant base layers (neck/wrist/ankle) and improved glove materials address laceration risk. Goaltender equipment dimensions are tightly regulated to balance safety and fairness [187].

Beyond personal equipment, venue infrastructure, such as flexible glass and compliant board systems, is associated with substantially lower injury risk at the elite level [188]. Independently, IIHF‐prescribed lighting standards (uniformity/CRI) are intended to improve puck/teammate visibility and officiating accuracy [189]. (Performance‐tuning elements of sticks, skates, and steel profiling are covered under “Evolution of Equipment”).

Assessing aspects of the competitive venue, e.g., ice temperature, hardness/roughness proxies, and wind in multi‐rink complexes, informs resurfacing and maintenance protocols [161]. Reliable radio communications and, in venues where available, 5G/backhaul, support coordinated medical response in accordance with official medical guidance [189, 190]. Emerging technologies are further refining safety protocols. Instrumented mouthguards (iMGs) and select wearables are emerging to quantify head‐ and knee‐load exposures. This data supports evidence‐based updates to rules and equipment, with growing validation from laboratory and on‐ice studies, including research in women's professional hockey [191, 192].

#### 6.2.4 Figure Skating

Although perceived as elegant and low‐impact, figure skating imposes substantial mechanical loads, especially during multi‐rotation jumps, where simulated landing trials report peak vertical ground‐reaction forces around five times body weight, with higher values depending on technique and jump height. Consequently, injury patterns are dominated by overuse injuries of the lower back, hips, knees, and ankles, while acute trauma occurs most often during jump take‐offs and landings [193, 194, 195].

Protective strategies therefore focus on equipment engineering, targeting both impact attenuation and load distribution. Boot stiffness and fit are scaled to athlete mass, strength, and jump repertoire to provide sufficient ankle support while preserving the dorsiflexion needed to absorb landing forces and the plantarflexion required for propulsion and take‐off [196, 197]. Simultaneously, blade geometry (including radius of hollow, rocker curvature, and toe‐pick configuration) is tuned to influence edge bite, effective contact length, and control of the spin axis. Poorly matched setups can increase shear at take‐off and impact loading at landing [98]. Blades with integrating damping reduce measured landing loads in simulated protocols without compromising jump height, illustrating a pathway for equipment‐level mitigation of overuse risk. These developments sit within ISU equipment rules that define permitted boot and blade characteristics to ensure fair competition and safe equipment configuration [83, 198].

#### 6.2.5 Curling

Falls on ice dominate the acute injury burden, particularly in recreational and novice settings, where head impacts and wrist/upper‐limb injuries are most common [199]. In competitive cohorts, by contrast, self‐reported problems skew toward overuse pain in the knee, back, and shoulder, with relatively low time‐loss rates [200]. Accordingly, basic personal protection centers on footwear and fall prevention: a gripper sole on the non‐sliding foot (and grippers when walking on the sheet), delivery stabilizers for beginners, and head‐protection policies in youth and learn‐to‐curl programs [111, 188, 201].

Venue preparation contributes directly to safety and consistency. Standard ice‐making and maintenance practices, such as pebbling (and subsequent surface finishing) and disciplined control of ice‐shed conditions, aim to deliver predictable glide while reducing slip‐trip hazards around hacks and along paths of play [202]. Day‐to‐day operations rely on trained ice technicians and clear procedures, supported by national training pathways [203] and the World Curling Federation's facility and competition standards [204]. Increasingly, rinks instrument their environments (e.g., logging ice and air temperature and humidity) to verify that the prepared surface remains within safe, performance‐appropriate ranges across busy schedules [205].

## 7 Data Acquisition Systems: Capturing the Athlete‐Environment Interaction

The modern Winter Olympic athlete is a connected athlete. Preparation and performance analysis now rely on a sophisticated ecosystem of sensors that capture physiological and biomechanical data in the field. The overarching challenge is a direct confrontation with environmental volatility, as teams must deploy sensitive electronics in hostile environments characterized by extreme cold, moisture, and high‐frequency vibrations.

Some systems are designed for athlete performance and feedback, while others serve audience‐facing purposes, such as enhancing broadcast graphics or providing real‐time statistics. Although both rely on similar sensing infrastructure, their validation pathways and ethical requirements differ substantially.

The cornerstone of modern field‐based biomechanics is fusing external tracking and kinematics. In practice, this is most commonly achieved through a combination of GNSS, often using high‐precision variants like RTK [206, 207] or dGNSS [208], and IMUs [22, 124, 179, 209]. This sensor fusion allows for the reconstruction of an athlete's full‐body 3D trajectory over an entire course, resolving critical performance variables like velocity profiles, turn radii, and line choice relative to the optimal path, as well as technique parameters [4, 21, 22, 209].

In cross‐country skiing, the same fusion quantifies technique use and transition timing (e.g., double‐poling vs. diagonal stride; skating gears 2/3 over terrain), enabling micro‐pacing analysis [122, 124, 179, 208, 210]. In alpine skiing, the analytics are complemented by instrumented equipment, such as strain‐gauged poles, pressure insoles/boot sensors, and force‐sensing bindings, to expose propulsion magnitude and timing, force‐application asymmetries, and impact loads [211]. Looking ahead, sensor‐equipped skis demonstrate on‐snow measurement, torsion and vibration content connected to ski performance, as well as a tool for ski selection and individualization of ski properties [212, 213, 214, 215].

Although biomechanical analysis has traditionally dominated winter sports research, sensor‐based physiological monitoring is increasingly being used to quantify internal load. The most prominent example is heart rate, which informs training intensity distribution and supports individualized training prescription [216]. Further insights into autonomic function can be obtained from heart rate variability (HRV). In endurance athletes, adjusting training based on HRV analysis has been associated with improvement of some physiological outcomes [13]. Additionally, in elite Nordic skiers following a “live high, train low” regimen, daily training loads adjusted using morning HRV measurements mitigated the typical decrease in parasympathetic activity observed with this training method [217]. Other relevant technologies include non‐invasive infrared thermography to assess muscular load, and wearable sensors to monitor sleep and optimize recovery [218, 219].

Portable metabolic systems for field measurement of oxygen consumption (VO_2_) are used to profile skiing economy and substrate utilization, but environmental factors (cold, humidity, wind) can affect sampling and sensors; therefore, windshields, air‐drying units, and on‐site validation are recommended (See Section 10) [220].

Major international winter‐sport competitions often require athletes to travel across time zones, adjust to unfamiliar environments, and, in some cases, compete at higher altitudes. These factors can contribute to sleep disturbances through mechanisms such as jet lag, travel fatigue [221], changes in ambient oxygen content, or unfamiliar sleeping environments, including the well‐known “first‐night effect” [222]. Managing these disruptors is critical for achieving peak performance during the Winter Olympics [223]. While different interventions can be pursued to counteract sleep disturbances, objective monitoring of an athlete's sleep using wearable technology, such as smartwatches or rings, is gaining increasing interest.

Technological advancements have narrowed the gap between research‐grade sleep monitoring in clinics and commercially available sleep trackers, but differences among specific devices and across sleep parameters still exist [218, 224]. Most commercially available wearables use a combination of sensors to assess parameters such as activity, heart rate, and heart rate variability to estimate sleep metrics [225].

While differences between devices exist to monitor different aspects of sleep, a recent study compared six consumer wearables against laboratory‐based polysomnography (PSG) and found that all tested devices had high overall agreement (≈86%–89%) with PSG when classifying epochs as sleep or wake, but only had moderate agreement (≈50%–65%) for distinguishing between specific sleep stages [226]. Despite this limitation, it was argued that wearables may assist in objectively determining the effect of travel fatigue and jet lag and support jet lag minimization strategies [218].

Conversely, other emerging sensor technologies often lack sufficient validation despite aggressive marketing. One example is the use of continuous glucose monitors (CGMs), which are promoted for supporting fueling strategies, avoiding glycogen depletion, and managing (rebound) hypoglycemia, as well as adjusting training loads. A recent article critically evaluated the use of CGMs in this context and emphasized key limitations, including concerns about measurement validity, the inherent temporal lag, the inability of CGMs to quantify overall carbohydrate availability, and the potential for misinterpretation by athletes and coaches [227]. Consequently, selecting, implementing, and applying the right technology at the right time remains crucial (see also section “Governance and Quality Control”).

## 8 From Data to Decision: Analytical Workflows and Performance Intelligence

This section focuses on performance technologies that transform raw sensor data into insights for training, coaching, or (medical) decision‐making. The raw‐data streams generated by these sensor systems are, on their own, of little value. Their potential is only unlocked when they are processed through a robust analytical pipeline designed to transform them from numbers into actionable insights and decision support for coaches and athletes. The objective of performance analytics is not just retrospective analysis, but prospective guidance: identifying precisely where, when, and how to modify aspects of training such as intensity, technique, tactics, or equipment to improve performance and mitigate injury risk.

Crucially, even quality‐controlled data streams are a starting point, not an endpoint. Decision‐making can be conceptualized as a hierarchy that progresses from raw data to actionable decisions [228], as cited in [229]. This framing clarifies the analytical and interpretive gap that remains after measurement: for example, dGNSS can show that a skier lost time in a turn [230], but not whether the cause was a technical error, inadequate muscle force production, or a tactical choice. At present, that contextual judgment rests with the coach and athlete. Technology can reveal patterns and possibilities, but its outputs still require human interpretation. Accordingly, understanding the division of data processing between the sensor's algorithm and the human user (coach or athlete) is crucial for the proper selection and application of these technologies [14].

A successful analytical workflow follows a clear path from data to decision [231]. It begins with time‐synchronized data acquisition from multiple sources (e.g., video, GNSS, IMU, environmental sensors) [23, 29, 31, 232]. This is followed by crucial quality control steps to address issues such as data dropouts, sensor drift, and latency. The cleaned data are then analyzed, and where raw signals are converted into meaningful performance metrics, such as turn radius, edge‐angle proxies, section split costs, start impulse metrics in alpine skiing, or indices of glide efficiency in cross‐country skiing. These features then feed into modeling, ranging from simple descriptive statistics to more complex predictive or prescriptive models [210].

**FIGURE 2:**
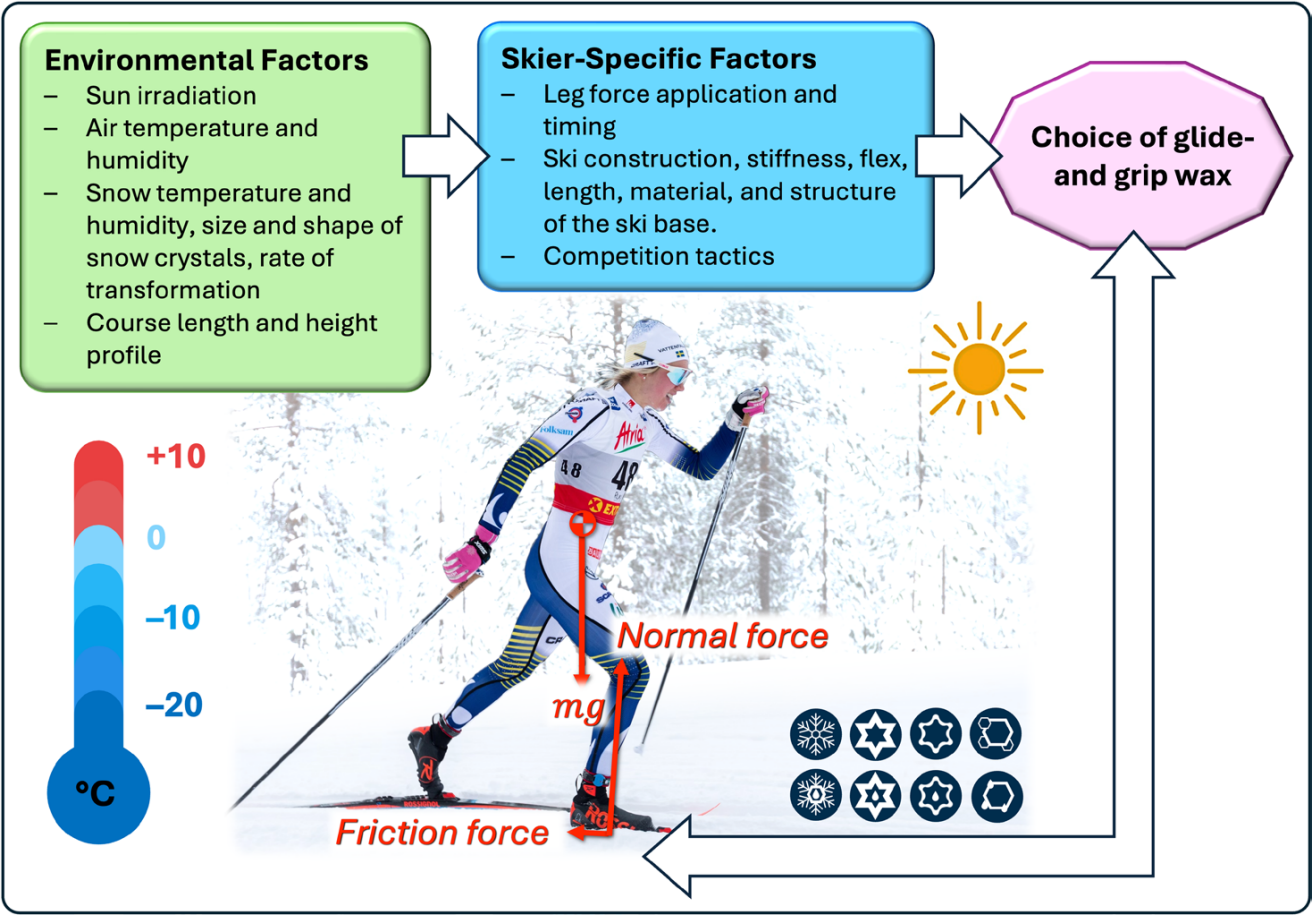
Environmental parameters (e.g., temperature, humidity, solar radiation, and snow‐crystal state) and skier‐specific factors (e.g., force application, equipment properties, and tactical choices) jointly determine the appropriate selection of glide and grip wax. The lower panel illustrates the tribological interface, highlighting the normal and friction forces acting on the ski and the influence of temperature and crystal morphology on frictional behavior.

The final step is communication, in which analytical outputs are translated into simple, intuitive, and coach‐ready decision support. To illustrate this translation, Figure 2, provides one example of how environmental and mechanical information can be synthesized into practical guidance for the selection of glide and grip wax. Similar communication principles underpin other forms of decision support, such as video overlays comparing an athlete's line with a faster competitor's, heat maps of time loss on a course, or key performance indicators reported with associated uncertainty bands [233].

The power of this approach is illustrated by canonical use‐cases across different sports. In alpine skiing, for instance, the combination of GNSS and synchronized video is used to quantify the mechanisms of time loss for each turn, relating the skier's line choice to the forces they experience and their ability to preserve speed through the turn's apex [156, 234]. This enables coaches to provide athlete‐specific advice online for different types of gate combinations [4].

In cross‐country skiing and ski mountaineering, GNSS profiles of a course are used for analyzing performance and pacing patterns in a race, including “micro‐pacing” audits, analyzing how an athlete distributes their effort on climbs, descents, and transitions, and to diagnose issues with glide performance under shifting snow conditions [9, 208, 210, 235]. In sliding sports, split‐to‐split velocity analysis is used to tie the initial start impulse and subsequent steering inputs to down‐track performance, helping teams optimize both the athlete's start and the sled's setup [89]. In ice hockey, emerging IMU‐based pipelines for individualized stick tuning have shown on‐ice feasibility, but should be independently validated on rink‐temperature data with quantified agreement, reliability, and uncertainty before routine adoption [236].

The ultimate goal is to close the feedback loop by linking analytical insights to concrete, coachable levers, for example, “On this pitch, your edge angle is consistently 5 degrees too low and lasts too long, costing you 0.05 s per turn; we need to work on maintaining higher inclination at the apex.” By logging the changes made in response to such feedback and tracking their subsequent outcomes, teams can continuously refine their analytical models and improve their decision‐making throughout the Olympic cycle [21, 31, 232].

## 9 Digital Arenas, AI, and Broadcasting Technology

The “digital arena” is the integration of real‐time data capture, visualization, and feedback directly into the competition environment, serving coaches, officials, broadcasters, and fans [24, 29, 31, 235, 237]. While broadcast‐oriented sports technology aims to enhance spectator engagement through real‐time rendering, virtual overlays, and immersive replays, it also serves as performance technology for coaches and athletes through live feeds from tracking systems that support immediate situational awareness in mass‐start formats and rapid between‐run review.

In alpine skiing, calibrated gate triggers, split‐timing systems, and radar guns power live TV graphics, while high‐precision GNSS (RTK or dGNSS) and IMUs are used by teams and researchers to reconstruct section speed, turn geometry, and run‐time differentials for rapid post‐run review. In addition, in pilot deployments at select venues, they provide for near‐real‐time overlays [22, 118, 209, 234]. For officiating, technology now routinely augments decision‐making: high‐resolution timing and tracking are standard in cross‐country skiing and biathlon [72, 238]. In curling, hog‐line sensors are mandatory and stone‐trajectory/“smart broom” systems are increasingly trialed [110, 111, 239, 240]. Multi‐angle video review is standard in short track and widely used in ice hockey and figure skating [83, 161], whereas in long‐track photo‐finish and false‐start systems remain primary, with video review applied as needed.

A major development area is AI‐assisted judging and performance analysis. Computer‐vision tools are being piloted to quantify rotations and landing quality in figure skating and freestyle skiing, providing objective cues to support human panels [99]. In ice hockey, player‐ and puck‐tracking streams feed machine‐learning models for tactical patterning and advanced metrics used by teams and broadcasters [94]. These systems are designed to augment, not replace, human officials, a model that necessitates clear validation standards and robust human‐in‐the‐loop procedures (see Section 10).

For broadcasters and fans, the same pipeline enables richer storytelling: synchronized trajectory overlays, real‐time velocity, and split‐time maps that reveal where time is gained or lost [21, 31, 232, 237]. This ecosystem is underpinned by purpose‐built venue networks, such as private 5G/edge architectures, capable of low latency, high bandwidth transport for multi‐camera video, sensor data, and graphics [28, 241]. Done well, the digital layer simultaneously elevates coaching feedback, officiating transparency, and spectator engagement.

However, for this technological ecosystem to be truly effective and equitable, it cannot be a monolith. While these systems offer universal benefits, their optimization demands a more granular, user‐centered approach. To unlock the full performance potential of every competitor, all preceding topics, from the material science of equipment and the engineering of venues to the logic of analytical models, must be viewed through a crucial and differentiating lens: sex‐specific adaptation [242, 243].

## 10 Governance and Quality Control

Ensuring that sport and performance technologies are safe, fair, and trustworthy requires explicit governance frameworks and rigorous quality assessment protocols. Federations and other stakeholders can support coaches and athletes in this process, reducing redundant work across winter sports disciplines. As the boundary between engineering innovation and athlete welfare becomes increasingly blurred, quality control (including, e.g., the assessment of validity) and governance must evolve in parallel with the technology itself.

The growing reliance on technologies, that sense and analyze different aspects in winter sports, demands that these technologies be controlled for their quality before influencing, e.g., aspects of training or competition, or public broadcasting. Assessing the quality of sports technologies can include a variety of aspects, including, e.g., validity, repeatability, usability and user experience, ethics and security (privacy, ownership, transparency), data management (standardization/interoperability), and environmental sustainability [244]. Within this governance structure, it is essential to distinguish athlete‐centered performance technologies (e.g., injury‐risk models, load dashboards) from audience‐facing sports technologies (e.g., broadcast overlays, venue graphics), as the former have direct consequences for fairness, health, and safety, and thus require stricter evidence and ethical standards.

A foundation of good governance is transparent data stewardship and informed athlete consent. Teams and federations should specify what data are collected, for what purposes (e.g., coaching, medical, research, broadcast), who owns them, and how they are accessed, shared, and retained. These policies must comply with existing laws such as the GDPR [245], and the forthcoming EU AI act. In addition, athlete sovereignty must be protected, and informed consent (which can be revoked) must be obtained after informing the athlete, especially where public dissemination is involved. Privacy‐by‐design principles, including data minimization and role‐based access control, should be standard practice [246].

Rigorous in situ validation is essential before any sensor or data acquisition system is used to inform training or competition decisions. Devices used in training or competition should be evaluated for accuracy, reliability, and responsiveness under realistic on‐snow or on‐ice conditions, not merely in laboratory settings [4, 23, 26]. Failure to validate has produced misleading feedback; for example, a sensor that performs perfectly at 20°C in a laboratory may provide dangerously erroneous data at −15°C on a vibrating downhill ski [247]. Optical HR/HRV wearables often exhibit temperature‐dependent drift, while IMUs may exhibit bias and scale errors due to thermal and vibrational loading.

Pose‐estimation pipelines can also display sex‐linked classification errors. Because anthropometric and biomechanical characteristics differ between sexes, sensor accuracy and calibration requirements may not be uniform. Validation datasets should therefore be sex‐balanced, and calibration procedures must account for these differences to avoid systematic measurement bias. Together, these considerations further underscore the risk of unverified deployment [247, 248, 249, 250].

Validation protocols should report temporal resolution, spatial precision, inter‐unit variability, and robustness to environmental noise, following standard reliability frameworks [251, 252, 253]. Known failure modes, such as photoplethysmography signal collapse during cold‐induced vasoconstriction or temperature‐dependent IMU drift, must be identified and mitigated through appropriate placement, attachment, and thermal calibration [247, 248]. Evidence from field VO_2_ testing in cross‐country skiing further demonstrates the feasibility of discipline‐specific validation under authentic outdoor conditions [254]. Despite the proliferation of consumer wearables, independent peer‐reviewed validation in elite winter‐sport contexts remains scarce [29, 255, 256], underscoring the need for standardized, transparent testing under the full range of environmental and mechanical stresses encountered in snow and ice competition.

Guidance for assessing the quality of various technologies is available in the literature [244, 248], and consensus frameworks for injury and illness surveillance in snow sports have been established [257]. Quality assessment strategies from football's *Electronic Performance and Tracking Systems* (EPTS) also provide transferable strategies for accuracy, latency, and reliability [258] or from World Rugby regarding the use of instrumented mouthguards to assess concussion [191, 259].

For AI‐based systems, whether predicting injury risk, modeling a specific technique, or assisting judges, the requirements for quality assessment are even more stringent. Predictive models must be trained on transparent ground‐truth labels and tested on independent datasets from different seasons, venues, or athlete populations to ensure generalizability and detect domain shift (i.e., distributional differences between training and deployment data) [260, 261]. Trust in model outputs depends on *explainable AI*, i.e., methods that make algorithmic reasoning interpretable to humans. Systems that justify their predictions are more likely to be trusted and acted upon by certain populations [262].

To maintain integrity over time, AI systems require lifecycle governance, including version control, periodic retraining, monitoring for performance drift, and rollback procedures in the event of degraded accuracy. Moreover, performance metrics should be disaggregated by athlete subgroups (e.g., sex, age, body size) to expose and mitigate bias [263, 264]. Machine‐learning models trained on sex‐imbalanced datasets risk encoding biased decision rules, potentially distorting injury‐risk predictions, technique assessments, or performance modeling. Accordingly, AI‐based systems require sex‐specific performance reporting and, where relevant, sex‐sensitive model calibration. Finally, because winter sports operate under unique physiological and environmental constraints, quality assessment protocols must reflect these specific demands [265].

Building strong governance and quality control frameworks is not an endpoint but the foundation for what comes next. As innovation accelerates, ensuring that new technologies are safe, sustainable, and ethically deployed becomes central to the future of winter sports. The next section explores how emerging materials, energy systems, and digital tools are redefining both performance and sustainability, alongside the ethical and operational responsibilities that follow.

Collectively, practical recommendations include:

− Establishing explicit governance frameworks and rigorous quality assessment, coordinated across federations to avoid redundant validation efforts.
− Transparent data governance specifying purpose, ownership, access, sharing, and retention, compliant with existing regulations such as the EU AI Act.
− In situ validation under realistic cold‐weather and high‐vibration conditions.
− Explainability of AI‐derived outputs to ensure interpretability and increase trust.

## 11 Future Directions: Innovation, Sustainability, and Ethical Considerations

The evolution of technology in winter sports is poised to accelerate across several converging fronts. Smart materials and adaptive equipment are expected to shift the paradigm from static, condition‐specific gear toward dynamic systems that respond in real time. For instance, future ski bases may integrate micro‐actuators that adjust surface texture in response to sensor‐detected snow morphology, while bobsleigh runners could employ thermoelectric elements to regulate ice‐interface temperature for optimal friction.

### 11.1 Technological Convergence and Design

Innovation will increasingly blur the boundary between spectator‐facing (broadcast) and athlete‐facing (performance) technologies, creating platforms that enhance both athlete output and the audience experience. Enabling technologies include soft robotics, bioinspired sensors, and functional composites [266, 267]. These may be coupled with triboelectric nanogenerators (TENGs) to power embedded sensors and actuators without bulky batteries, an especially valuable feature for cold, remote venues where traditional power sources are unreliable [268, 269, 270]. Naturally, all such innovations must comply with sport‐specific regulatory frameworks of the respective sport, even as these rules evolve to accommodate new technologies.

The design cycle for next‐generation equipment will be further accelerated by AI‐driven design pipelines and digital twins, enabling engineers to simulate and optimize thousands of prototype iterations in silico before physical production. These virtualized workflows promise unprecedented personalization, that is, equipment tuned to individual athletes, venues, and environmental conditions, while reducing cost and material waste.

### 11.2 Sustainability as a Core Tenet

This pursuit of performance must be balanced with an equally strong commitment to sustainability. Priorities now extend beyond fluor‐free waxes to include AI‐optimized snowmaking systems that minimize energy and water use, circular material life cycles for skis and boots through remanufacturing and recycling, and biodegradable structural composites. These directions align with the Olympic Movement's sustainability goals and the growing recognition of sport's climate footprint [271, 272]. The integration of self‐powered sensing through TENGs provides an additional sustainability pathway by reducing dependency on disposable batteries and external power infrastructure [270].

### 11.3 Governance, Ethics, and the “Zone of Uncertainty”

The rapid integration of technology compels the winter‐sports community to confront ethical and equity challenges. Governance structures must address the use of real‐time biometric data, especially in public broadcasts or AI‐based performance analytics [273]. Equally, the accelerating pace of innovation risks widening the gap between resource‐rich and smaller federations. To counter this, international bodies should pursue technology‐solidarity initiatives. These include open‐access data repositories, shared testing infrastructures, and collaborative design networks, ensuring that innovation enhances, rather than undermines, global competitive integrity [40, 274].

As technologies increasingly shape elite winter sports, future governance must determine where to impose restrictions. In this matter, a recent debate has emerged, for example, in running, where footwear technology affects running performance, yet no definitive conclusion has been reached [275, 276, 277]. As technologies become more readily available, such discussions are increasingly necessary in winter sports.

At the same time, innovation should not sterilize the essence of winter sport. The appeal of competition rests on fair contest within managed variability; if measurement and control remove too much uncertainty, events become more predictable and less compelling for athletes and spectators alike. Governance should therefore protect a deliberate “zone of uncertainty”, for example, limit in‐race telemetry that dictates tactics, avoid over‐standardizing surfaces beyond safety, and ensure analytics inform rather than prescribe competitive decisions.

As technological innovation accelerates, the challenge is no longer whether new tools can enhance performance, but whether their use aligns with principles of fairness, safety, and sustainability. The following Section 12 reflects on this balance, emphasizing the shared responsibility among engineers, scientists, coaches, and governing bodies to ensure that innovation strengthens the integrity of the Winter Games.

Looking ahead, several developments are likely to shape the technological landscape of winter sports. Collectively, these involve progress not only in engineering tools but also in sustainability, data governance, and equitable access. Ideally, future directions will include:

– Adaptive Systems: AI‐driven, sensor‐integrated equipment enabling personalization while reducing material waste
– Embedded Sustainability: Eco‐friendly materials, reusable or multi‐use components, efficient snowmaking, and circular product cycles.
– Energy Autonomy: Self‐powered sensing systems reducing ecological burden and infrastructure dependence.
– Data Ethics: Strict governance of biometric data, transparency, and athlete protection in data‐intensive environments.
– Validation Standards: Rigorous quality control for commercially available athlete technologies.
– Equity Access: Mechanisms for equity‐focused access to technology to prevent widening performance gaps and preserve fairness.

## 12 Authors' Perspective

The integration of sports and performance technologies is transforming how winter sports are performed, governed, and experienced. Recognizing the duality between systems that enhance the competition experience and those that support athletic development is essential for fair and future‐proof innovation.

Smart materials, embedded sensors, energy‐harvesting systems, and AI‐based analytics now influence everything from friction management and technique feedback to equipment design and broadcast presentation. Yet these technologies only fulfill their potential when grounded in robust quality control, ethical deployment, and equitable access. Performance no longer depends solely on physical capacity and tactical skill but on the quality of interaction between athlete, environment, and technology.

Innovation alone does not guarantee progress. Low‐quality technologies can mislead coaches, disadvantage athletes, or compromise privacy and consent. Without sound governance and quality control, technical systems risk becoming opaque or exclusionary, while unregulated advances in equipment can widen disparities between nations.

As the Milano–Cortina 2026 Winter Olympics approach, winter sport stands at a decisive point. Pairing smart, sustainable equipment with transparent governance and quality control offers a path toward faster, safer, and fairer competition, where technology serves not only performance but the enduring integrity of sport itself.

## Funding

This research was partially funded by (i) the Swedish Agency for Economic and Regional Growth (grant no. 20369543) and (ii) the Slovenian Research Agency (grant no. P5‐0147).

## Conflicts of Interest

The authors declare no conflicts of interest.

## Data Availability

Data sharing is not applicable to this article as no datasets were generated or analyzed during the current study.
